# High-Sensitivity Solution-Processed Organic Phototransistor Based on a Bulk Heterojunction with a Persistent Radical as the Electron Acceptor

**DOI:** 10.1021/acsaelm.4c02334

**Published:** 2025-04-23

**Authors:** Giulia Baroni, Francesco Reginato, Sara Mattiello, Salvatore Moschetto, Mario Prosa, Margherita Bolognesi, Luca Beverina, Stefano Toffanin

**Affiliations:** † Institute of Nanostructured Materials (ISMN)—National Research Council (CNR), Via P. Gobetti 101, Bologna 40129, Italy; ‡ Department of Materials Science, University of Milano-Bicocca, Via R. Cozzi 55, Milano 20126, Italy

**Keywords:** solution-processed organic phototransistor, radical acceptor, photogain, electron trapping, electron-only device, photosensitivity

## Abstract

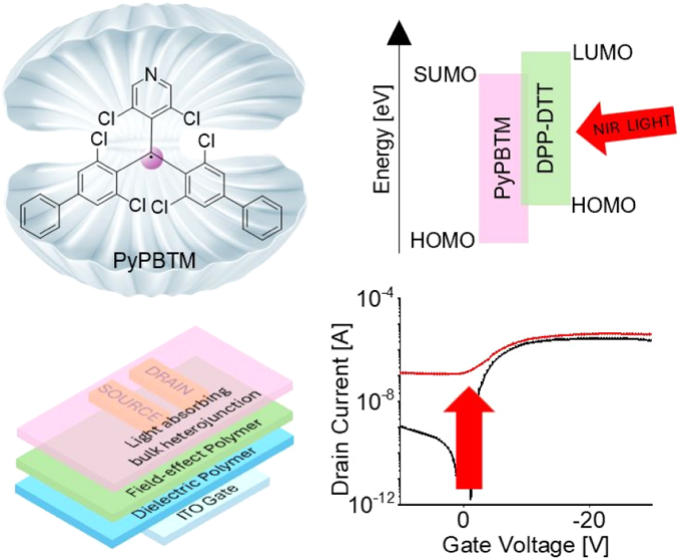

In bilayer organic phototransistors (OPTs), charge transport and light-sensing functionalities are separately performed and optimized in two different layers. For optimizing the sensitivity of solution-processed bilayer OPTs, the approach of using a donor–acceptor bulk heterojunction (BHJ) as the light-sensing layer is well established in the literature, but the choice of the electron-accepting materials is often limited to fullerene-soluble derivatives or to standard nonfullerene acceptors. Herein, we report the unprecedented use of an organic persistent radical as an electron acceptor in the BHJ light-sensing layer of solution-processed bilayer OPTs. The radical acceptor is coupled at different donor:acceptor ratios to a low-band-gap polymer that absorbs in the near-infrared (NIR) region. At a donor:acceptor ratio of 1:3, the organic radical forms isolated domains within the BHJ. Such a morphology, coupled with the strong electron-accepting characteristics of the radical, leads to efficient trapping of electrons and efficient hole transport within the BHJ, as measured in charge-selective devices operated in the space-charge limited current (SCLC) range. This, together with the chemical and photostability of the persistent radical, allows us to obtain an OPT with photosensitivity (*P*) of 1 × 10^5^ in response to NIR irradiation at 2 mW/cm^2^ and excellent photostability over time.

## Introduction

In recent years, organic phototransistors (OPTs) have attracted much interest owing to their capability of combining in a single device, both the functionalities of light detection of organic photodiodes (OPDs) and the typical signal amplification of organic field-effect transistors (OFETs) compared to other integrated or monolithic multicomponent approaches.^1^ In the future, OPTs sensitive to near-infrared (NIR) wavelengths are expected to become key components in optoelectronic integrated circuits for applications in energy storage and harvesting,^2^ optical communications and computing,^3^ as well as in the medical,^4,5^ augmented reality, and military sectors.^6−8^ Compared to their inorganic counterparts, solution-processed OPTs show favorable properties, such as lightweight, low fabrication costs, good biocompatibility, and tailored spectral response by chemical design.^9−11^ To overcome the limitations related to the low carrier mobility of single-layer OPTs, in recent years, bilayer OPT structures have been introduced, where field-effect transport is provided by an organic semiconductor thin film, while light absorption and exciton dissociation occur in a separated photoactive layer that is commonly stacked on top.^2^ The photoactive layer is typically a bulk heterojunction (BHJ) composed of an electron donor and electron acceptor material,^12^ providing absorption in the spectral region of interest.^13−15^ By separating the charge carrier transport and light absorption functions into different layers, it is possible to separately optimize the electrical and optical characteristics of the OPT.

Most often, in bilayer OPTs, the selection of the BHJ components is based on a large library of materials borrowed from BHJ organic photovoltaics.^16−18^ For BHJ photovoltaic applications, the acceptor material is typically a small molecule that (i) presents complementary spectral absorption with respect to the donor material, and (ii) can efficiently transport electrons within the BHJ along continuous domains that are finely intermixed with the domains of the donor material.^19^ Indeed, BHJs that are optimized for photovoltaic applications should present the electrical characteristics of well-balanced (and maximized) hole and electron mobilities.^20^

With respect to organic solar cells, which only operate in the photovoltaic mode, OPTs transduce the light signal into an electrical signal by the photogain (*G*) effect given by the photoinduced charges generated in the BHJ photoactive layer. In OPTs, *G* is directly proportional to the ratio between the lifetime of the photoinduced minority charge carriers and the transit time of the photoinduced majority carriers. Thus, *G* is also proportional to the ratio between the mobility of majority and minority charge carriers.^21,22^ Therefore, in OPTs based on a BHJ photoactive layer, retention or trapping of minority charge carriers is required for a large *G* and for an improved OPT performance.^23−25^

In this context, neutral open-shell molecules, such as organic persistent radicals, which display a singly unoccupied molecular orbital (SUMO) prone to accept and trap electrons,^26^ are expected to behave as highly performing electron acceptors in photoactive BHJs for application in OPTs. Organic persistent radicals are rapidly gaining attention for applications in optoelectronics because of some peculiar characteristics given by their electronic structure, such as their intrinsic ability to overcome spin statistic limitations for light emission, and they have been investigated both theoretically and experimentally.^27,28^ Recently, we have reported new applications of organic persistent radicals in optoelectronic components and devices, such as solar concentrators and light-emitting transistors.^29,30^ In the latter case, we clearly demonstrated that the increase of the efficiency of light emission is ascribed to electron trapping at the radical sites. To the best of our knowledge, this distinguishing feature of organic persistent radicals has not been directly exploited for obtaining efficient OPTs. Only a recent work reports the study of OPTs containing a light-absorbing BHJ doped with transient radicals, formed in situ under illumination by a photoinitiator.^31^

In this work, we present a new bilayer OPT comprising an (i) BHJ photoactive layer, based on an organic persistent radical as the electron acceptor and a low-band-gap polymer as the light-absorbing/electron donor, and (ii) a high-mobility hole-transporting semiconducting layer based on the same low-band-gap polymer.

In detail, the low-band-gap polymer is poly[2,5-(2-octyldodecyl)-3,6-diketopyrrolopyrrole-*alt*-5,5-(2,5-di(thien-2-yl)thieno [3,2-*b*]thiophene)] (DPP-DTT), and the organic persistent radical is (2,6-dichloropyrid-4-yl)bis[2,6-dichloro-(4-phenyl)phenyl]methyl (PyPBTM).

DPP-DTT is largely used in OPTs for its absorption properties in the NIR region, which makes it suitable for a plethora of applications ranging from the biomedical to the optical communication fields, and for its hole-transporting properties with hole mobility as high as 1 cm^2^ V^–1^ s^–1^, which enable its use in organic field-effect transistors (OFETs).^31,32^ DPP-DTT can also be cross-linked (cross-linker: 1,6-bis(trichlorosilyl)hexane or C6Si) and used as a solvent-resistant layer in full solution-processed multilayer devices.^6^

PyPBTM is a persistent open-shell molecule, whose interesting optical characteristics have already been reported in the recent literature.^29^ PyPBTM also presents remarkable photostability, as expected for other PyBTM-like radicals in comparison to other open-shell molecules. It is therefore well suited for light-sensing applications.^33^

To enable full solution processing of the device, the here proposed bilayer OPT architecture comprises also a solution-processed transparent dielectric, i.e., terpolymer poly(vinylidene fluoride-trifluoroethylene-chlorofluoroethylene) (PVDF-TrFE-CFE).^34,35^

The DPP-DTT/PyPBTM ratio of the BHJ photoactive thin films is varied, and the light absorption and morphological properties and the charge mobility characteristics of the resulting BHJs are studied and correlated with the performance of the NIR-sensitive OPTs in which they are incorporated, in terms of photosensitivity (*P*), responsivity (*R*), specific detectivity (D*), and time response.

## Experimental Section

### Materials

1,6-bis(trichlorosilyl)hexane (C6Si), PC_61_BM (*M*_w_ = 91,088 g/mol), PEDOT:PSS (P VP Al 4083), and organic solvents (cyclopentanone, chloroform, and chlorobenzene) were purchased from Sigma-Aldrich. DPP-DTT (*M*_w_ > 200 kDa) was purchased from Ossila. PyPBTM was synthesized according to a published procedure.^29^ PVDF-TrFE-CFE was purchased from Arkema. The solution for the hole-transporting semiconductor layer was prepared by dissolving the DPP-DTT polymer in C_6_H_5_Cl at a concentration of 4 mg mL^–1^, followed by stirring at 80 °C for complete dissolution. Then, a blend solution with 51 wt % C6Si was prepared by adding 1.6 μL of C6Si to 0.5 mL of a polymer solution and stirred at 80 °C overnight before spin-coating in a glovebox. Solutions of BHJ (10 mg/mL) were prepared by dissolving different ratios of D:A in chloroform, followed by overnight stirring at 55 °C. P(VDF-TrFE-CFE) (70:30:8,5) in the form of a powder was used to prepare the solution by dissolving 7 wt % polymer in cyclopentanone, followed by overnight stirring at room temperature. Zinc oxide (ZnO) used as an electron-injecting layer in electron-only devices was provided by Genesink. Glass indium tin oxide substrates were purchased from Visiontek System Ltd. All chemicals were used as received without further purification.

### Phototransistor Fabrication

**Figure 1:**
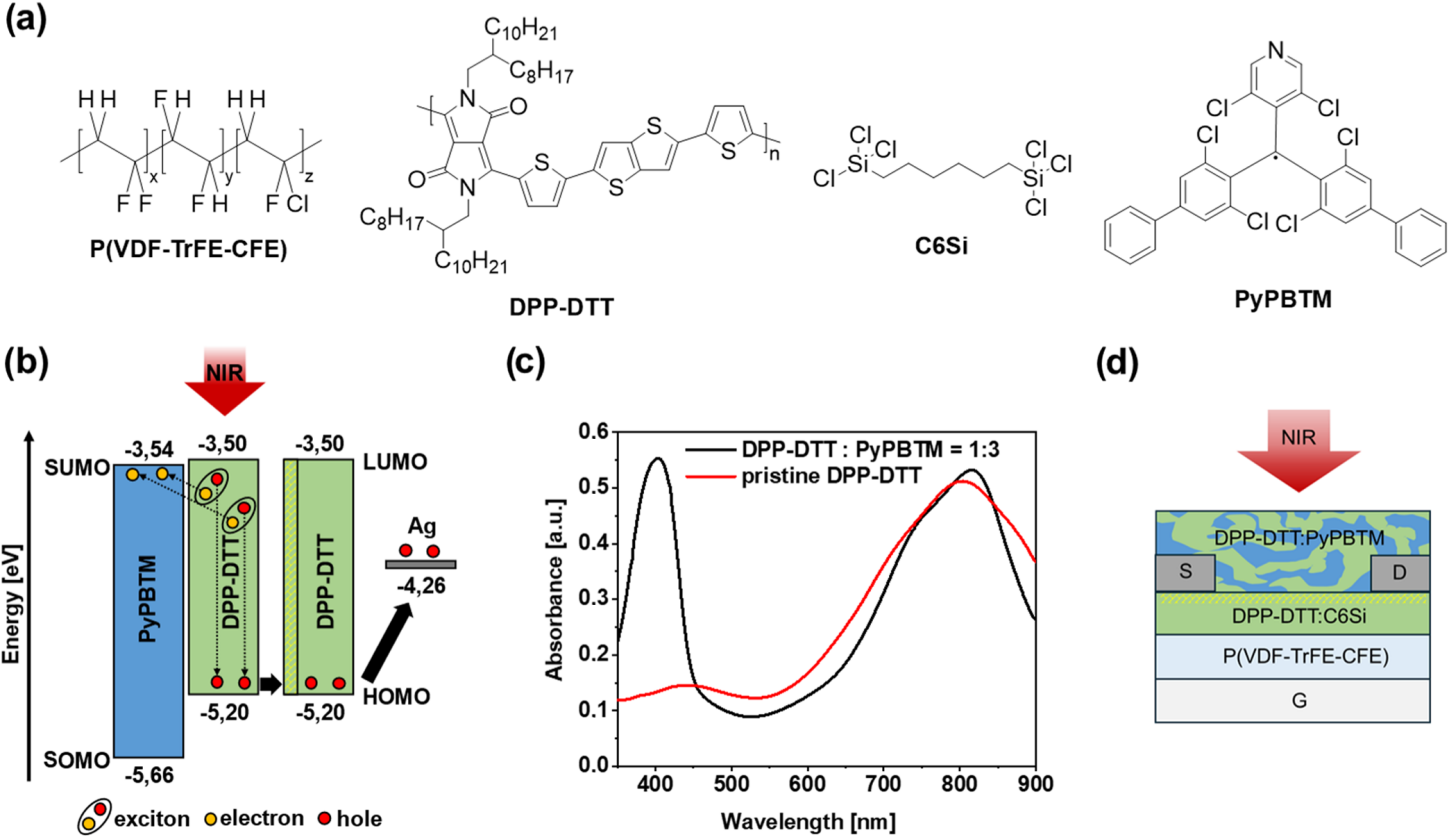
(a) Molecular structures of the materials used in the bilayer OPTs: dielectric material P(VDF-TrFE-CFE), hole-transporting DPP-DTT polymer, cross-linker C6Si, and PyPBTM electron acceptor. (b) Schematic energy level diagram of the BHJ donor and acceptor materials BHJ. (c) Absorption spectra of DPP-DTT/PyPBTM = 1:3 BHJ and pristine DPP-DTT thin films. (d) Cross-sectional view of OPTs with a bottom-gate middle-contact configuration.

A schematic description of the fabrication process for bottom-gate, middle-contact (BG-MC) transistors is shown in Figure 1b. First, glass/ITO substrates (25 mm × 25 mm) capable of locating 8 transistor devices were cleaned in an ultrasonic bath in acetone twice for 10 min, 2-propanol for 10 min, and then dried with argon flow. All substrates underwent surface treatment with oxygen plasma prior to polymer film deposition. The P(VDF-TrFE-CFE) (70:30:8.5) solution was filtered through a 0.45 m PTFE filter and then spin-coated at 4000 rpm for 90 s and annealed in a nitrogen atmosphere in a glovebox on a hot plate at 110 °C for about 30 min to remove the solvent (Ci = 76 nF/cm^2^ and thickness = 500 nm). Then, the DPP-DTT:C6Si solution was spin-coated on the dielectric at 1000 rpm for 60 s, after which the films were removed from the glovebox. After exposure to air for about 1 h, the polymer films were returned to the glovebox and annealed at 90 °C overnight and then at 200 °C for 10 min. The top contact source/drain electrodes were deposited by the thermal evaporation of Ag (70 nm thickness) using a metal shadow mask (100 μm channel length and 5000 μm channel width). Afterward, the BHJ blends were filtered through a 0.45 m PTFE filter, spin-coated at 1000 rpm for 60 s, and then placed on a hot plate for 10 min at 135 °C.

### Thin-Film Characterization

Ultraviolet–visible (UV–vis) absorption spectra of the BHJ thin films deposited on quartz substrates were obtained using a Jasco V-550 UV/vis spectrophotometer. The scanning electron microscopy (SEM) measurements were performed on DPP:DTT(cross-linked)/DPP-DTT:PyPBTM(1:3) thin films deposited onto silicon/native silicon oxide substrates, mechanically broken and mounted on a sample holder to observe the thin film section. Images were collected using an SEM ZEISS LEO 1530 FEG, with 5 kV electron beam acceleration.

### Phototransistor Characterization

The surface morphologies of the BHJ films in the OPTs were studied by atomic force microscopy (AFM). AFM imaging was performed in peak force mode on a Multimode 8 microscope (Bruker) equipped with a Nanoscope V controller, a type-JV piezoelectric scanner, and an air probe holder with SNL-A probes (Bruker). The lateral tip velocity was kept below 5 μm/s and forces below 500 pN were applied to minimize tip and sample wear. Background subtraction and image analysis were performed in Gwyddion 2.61. All OPT electrical measurements were performed under dark conditions inside a glovebox using a standard SUSS probe station coupled with a B1500A Agilent semiconductor device analyzer. The same equipment was used to measure the OPT characteristics under NIR illumination with an LED source (Thorlabs), which peaked at 770 nm with an intensity modulable from 2 mW/cm^2^ to lower values by using neutral optical density filters. The LED is driven using a Keithley 236 source measurement unit with a voltage input of 50 mA. The optical power density of the output illumination was measured using a calibrated Si-photodiode.

### Calculation of Electrical and Optoelectronic Parameters

The field-effect device mobility is calculated according to the following equation
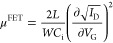
1where *L* is the channel length, *W* is the channel width, *C*_i_ is the capacitance per unit area of the gate dielectric, *I*_D_ is the source-drain current, and *V*_G_ is the gate voltage.

The performance of a photodetector can be characterized by three important parameters: responsivity (*R*), sensitivity (*P*), and specific detectivity (D*)
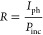
2
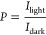
3
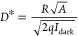
4where *I*_ph_ is the photocurrent, *P*_inc_ (= *L*_i_ × *A*, where *L*_i_ is the incident light intensity and *A* = 5 × 10^–3^ cm^2^ is the transistor channel area) is the optical power incident on the transistor channel, *q* is the elementary charge of the electron, and *I*_dark_ is the current under dark conditions. For the calculation of D*, shot noise was assumed to be the main source of dark noise.

### Fabrication of Electron-Only Devices (EODs) and Hole-Only Devices (HODs)

Glass/ITO patterned substrates (18 mm × 25 mm) capable of locating 6 devices were cleaned in an ultrasonic bath in acetone twice for 10 min, in 2-propanol for 10 min, and were dried with argon flow. All substrates underwent surface treatment with oxygen plasma prior to polymer film deposition. For the HODs, a PEDOT:PSS (≈40 nm) hole injection layer was spin-coated onto the cleaned substrates at 5000 rpm for 60 s and then placed on a hot plate for 10 min at 150 °C. For the EODs, a ZnO electron-injecting layer (≈40 nm) was spin-coated onto the cleaned substrates at 2000 rpm for 60 s and then placed on a hot plate for 5 min at 80 °C. For both HODs and EODs, BHJ solutions were filtered through a 0.45 μm PTFE filter and then spin-coated on both devices at 1000 rpm for 60 s, followed by 10 min on a hot plate for 10 min at 135 °C. The film thicknesses were measured using a profilometer (KLA Tencor, *P*6̅) as follows: DPP-DTT/PC_61_BM-CHCl_3_ = 85 ± 10 nm and DPP-DTT/PyPBTM = 1:3 = 90 ± 10 nm Then, Au (50 nm) and LiF/Al (0.6/100 nm) for HODs and EODs, respectively, serving as top electrodes, were deposited via thermal sublimation through a shadow mask onto the photoactive layer (active area of each device: 8 mm^2^).

### Calculation of Electrical Parameters from the Characteristics of HODs and EODs

The current density/voltage (*J*–*V*) curves of the HODs and EODs were collected in the range from 0 to 10 V to determine the space-charge limited current (SCLC) conditions and calculate the mobility (μ^SCLC^) of holes and electrons in the BHJ. This can be done by plotting the measured *J*–*V* curves as *Jd*^3^ vs *V*, and fitting the resulting curves with the Mott–Gurney equation
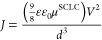
5where ε_0_ is the vacuum dielectric constant, ε is the film-specific dielectric constant, *d* is the BHJ film thickness, and *q* is the elementary charge.

## Results and Discussion

The chemical structures of PyPBTM and DPP-DTT, cross-linker C6Si, and dielectric PVDF-TrFE-CFE are shown in Figure 1a. Considering the energy level distribution of DPP-DTT and PyPBTM (Figure 1b), the photoinduced excitons in the DPP-DTT phase of the BHJ can be separated into charges by energetically favored electron transfer from the lowest-unoccupied molecular orbital (LUMO) of DPP-DTT (−3,50 eV) to the singly unoccupied molecular orbital (SUMO) of PyPBTM (−3,54 eV). In addition, holes can diffuse from the DPP-DTT in the BHJ layer to DPP-DTT field-effect layer owing to an isoenergetic level alignment.

Figure 1c shows the absorption spectra of a pure DPP-DTT thin film and DPP-DTT:PyPBTM = 1:3 BHJ thin film. DPP-DTT shows a strong absorption band centered at about 800 nm, while PyPBTM is responsible for the absorption at 400 nm. Therefore, upon irradiation of the BHJ with NIR light, photoinduced excitons are expected to form only in the DPP-DTT phase of the BHJ.

To enable efficient absorption by the BHJ photoactive layer, as reported for other transistor devices,^36^ a bottom-gate middle-contact configuration of the bilayer OPTs is chosen. In detail, the cross-sectional view of the radical-based OPT (r-OPT) in Figure 1d shows that the source and drain electrodes are embedded between the hole-transporting semiconducting DPP-DTT layer and the BHJ photoactive layer.

The composition and morphology of the BHJ should be optimized in a compromise between (i) the absorbance in the NIR by DPP-DTT to generate a large amount of photoexcitons in the donor phase, (ii) the suitable dispersion of PyPBTM within DPP-DTT to provide the interfacial area for photoinduced charge separation and trapping of minority charge carriers, thus minimizing charge recombination, and (iii) the interconnectivity between the donor and acceptor phases to allow the percolative pathways for the majority charge carriers toward the hole-transporting DPP-DTT layer. Collectively, these factors must be balanced to maximize *G* (and consequently *P*) in the bilayer OPTs comprising the BHJ photoactive layer. Here, we expect that *G* would be maximized when the lifetime of minority charge carriers (electrons) in the BHJ is maximized by trapping at PyPBTM sites, while a high mobility and short transit time are preserved for majority charge carriers (holes) diffusing within the DPP-DTT domains and contributing to the source-drain current flowing (mainly) in the bottom semiconducting layer.

The composition of the BHJ was varied by using three different DPP-DTT:PyPBTM weight ratios, keeping the total concentration of the components of the BHJ constant. Figure S1 shows the absorption spectra of the as-realized BHJ films with DPP-DTT:PyPBTM weight ratios of 1:2, 1:3, and 1:4. By increasing the PyPBTM content in the DPP-DTT:PyPBTM BHJ from 1:2 to 1:3 to 1:4 and preserving the BHJ film thickness at about 85 nm, the intensity of the absorption band at 400 nm increased, while the DPP-DTT absorption peak centered at 800 nm decreased in intensity, with the maximum absorption passing from 0.51 to 0.44 to 0.26. Therefore, at the same irradiation power density in the NIR range, a gradual decrease in the density of photoinduced excitons in the three BHJs is expected, passing from donor:acceptor weight ratios of 1:2, 1:3, and 1:4.

**Figure 2:**
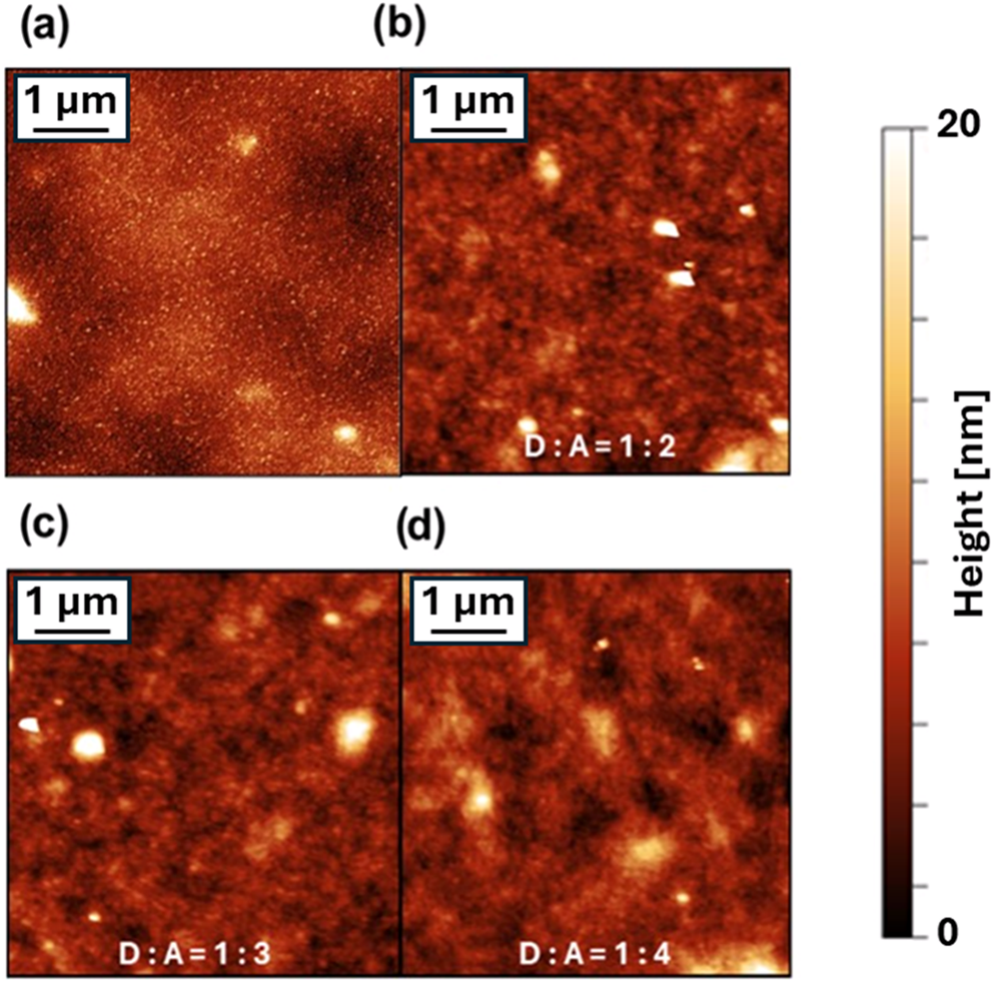
(a) AFM image of the pristine DPP-DTT layer. (b–d) Representative AFM images of the thin films of the three BHJs under study: (b) DPP-DTT:PyPBTM = 1:2, (c) DPP-DTT:PyPBTM = 1:3, and (d) DPP-DTT:PyPBTM = 1:4. All the films are processed onto the cross-linked DPP-DTT layer to mimic the morphology of the final multilayer OPT stack. All the images are 5 × 5 μm^2^ wide.

The morphologies of the BHJ films at donor:acceptor weight ratios of 1:2, 1:3, and 1:4, as well as the morphology of the pristine DPP-DTT film, were investigated by AFM, and the acquired images are shown in Figure 2a–d. A smoother morphology is observed in the case of the pristine DPP-DTT film, while a coarser morphology is observed in the case of the BHJ films. However, independent of the concentration, PyPBTM aggregates could not be clearly identified in the topographical images of the BHJ thin films. By considering the averaged root-mean-square roughness (*S*_*q*_) as a qualitative descriptor of the morphology of the three BHJs, we observed that *S*_*q*_ increased slightly, passing from 1.2 ± 0.1 to 1.4 ± 0.1 to 1.7 ± 0.1 nm, with increasing ratio of the DPP-DTT:PyPBTM components. This may be correlated with a gradual increase of the size and/or segregation degree of the PyPBTM domains within the DPP-DTT polymeric matrix.

**Figure 3:**
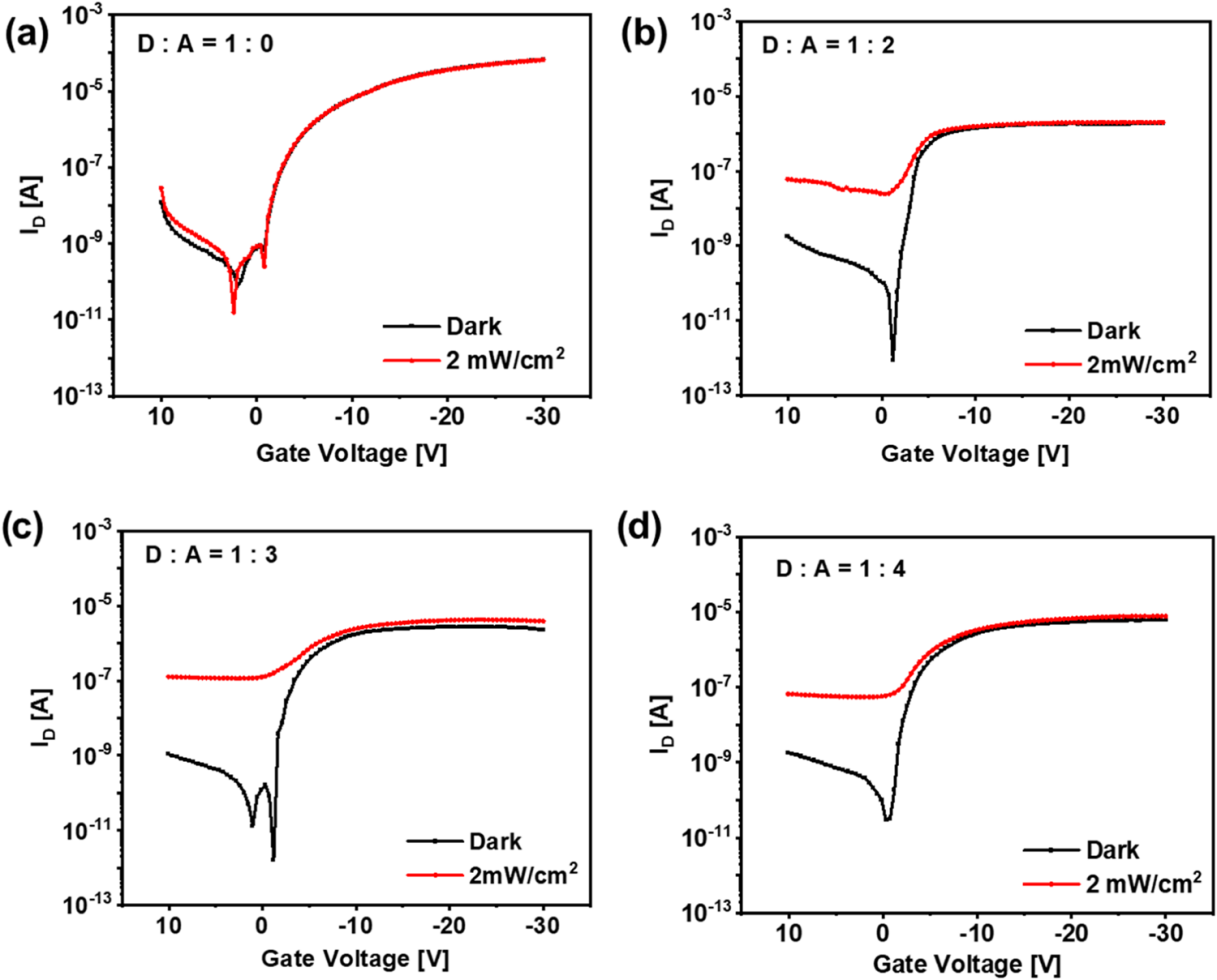
Transfer curves at *V*_D_ = −8 V in the dark and under 2 mW/cm^2^ of NIR light. (a) The reference DPP-DTT-only OPT (b–d) of OPTs based on BHJs under study with different DPP-DTT:PyPBTM weight ratios: (b) DPP-DTT:PyPBTM = 1:2, (c) DPP-DTT:PyPBTM = 1:3, and (d) DPP-DTT:PyPBTM = 1:4.

BHJ thin films with different donor:acceptor weight ratios were included as light-absorbing layers in complete radical-based OPTs (r-OPTs). The transfer curves of the different OPTs at a fixed *V*_DS_ bias of −8 V collected both under dark conditions and under NIR light (770 nm) are shown in Figure 3, and the electrical and optoelectronic figures of merit (FOMs) of the devices extrapolated from those curves are reported in Table 1, together with the FOMs of the reference OPT having a bilayer architecture with the acceptor-free photoactive layer, i.e., composed of DPP-DTT only (Figure S2). Under the dark conditions, all r-OPTs have field-effect hole mobility (μ_P_^FET^) of about 2 × 10^–2^ cm^2^ V^–1^ s^–1^, low threshold voltage (|*V*_TH_| < 3 V), and low loss currents at the gate electrode (on the order of hundreds of nA). The preservation of the OFET performance in the dark of the r-OPTs with respect to the DPP-DTT-based OFET, confirms the robustness of cross-linked DPP-DTT and of the PVDF-TrFE-CFE dielectric layers toward the subsequent solution processing of the BHJ photoactive layers on top, in agreement with our previous results.^35^

**Table 1:** Summary of the FOMs of r-OPTs and of the Reference DPP-DTT-Only OPT

DPP-DTT:PyPBTM	μ_P_^FET^^a^ [cm^2^/V·s]	*V*_TH_^a^ [V]	*P*^b^	*R*^b^ [A/W]	D*^b^ [Jones]
1:0	2.1 × 10^–2^	–2.63			
1:2	2.1 × 10^–2^	–1.23	3.6 × 10^4^	0.02	5.1 × 10^12^
1:3	2.1 × 10^–2^	1.50	1 × 10^5^	0.16	1.9 × 10^13^
1:4	2.1 × 10^–2^	0.64	2.0 × 10^3^	0.14	1.6 × 10^12^

a Calculated from the transfer curves in the saturation regime under dark conditions.

b Calculated from the transfer curves at *V*_DS_ = −8 V; the best results are reported.

The presence of PyPBTM as an electron acceptor in the BHJs of r-OPTs enables photoinduced charge separation in the BHJ, leading to a photoresponse to NIR irradiation of the devices at 770 nm (Figure 3b–d). However, the OFET based on pristine DPP-DTT does not show a measurable response to NIR (Figure 3a). In detail, the transfer curves of the r-OPTs collected under dark and NIR illumination (Figure 3b–d) exhibit typical OPT behavior: (i) at a gate voltage (*V*_G_) in the subthreshold region (*V*_G_ < *V*_TH_), the drain current *I*_D_ under illumination is much larger than *I*_D_ under dark conditions, owing to the photovoltaic and photogain effects, and *P* is maximized; (ii) at *V*_G_ > *V*_TH_, the device is in its on-state and the difference between *I*_D_ in dark and under illumination is inferior because the number of photoinduced charges in the BHJ is lower than that of the majority charge carriers already flowing in the bottom semiconducting layer. In this *V*_G_ range, *R* is maximized.^10,37^

*R* is more dependent on the electrical and geometrical OFET characteristics rather than on the type of BHJ.^2^ However, the best *R* = 0.16 A/W is reached for the 1:3 DPP-DTT:PyPBTM BHJs. More relevant differences within the r-OPTs are observed in terms of *P* and D*. *P* increases by one and 2 orders of magnitude when passing from the 1:2 or 1:4 DPP-DTT:PyPBTM BHJs to 1:3, respectively. *P* reaches a value as high as 1 × 10^5^ for r-OPT with 1:3 DPP-DTT:PyPBTM BHJ. Maximum values of D* of about 5.1 × 10^12^ and 1.9 × 10^13^ Jones, respectively, are found for the r-OPT comprising DPP-DTT:PyPBTM BHJ with the 1:2 and 1:3 weight ratios. Instead, D* decreases to 1.6 × 10^12^ for the r-OPT comprising the DPP-DTT:PyPBTM BHJ with a 1:4 weight ratio.

The observed trends of *P* and D* suggest that the r-OPT comprising DPP-DTT:PyPBTM BHJ with a 1:3 weight ratio provides the best compromise between exciton formation in DPP-DTT, photoinduced charge separation at the PyPBTM and DPP-DTT interfaces, minority charge carrier trapping, and majority charge carrier percolation to the bottom DPP-DTT semiconducting layer.

Probably, while the best compromise is reached by increasing the content of PyPBTM in the BHJ from 1:2 to 1:3, a further increase (from 1:3 to 1:4) may perturb the structural organization of the DPP-DTT domains, thus preventing the holes from diffusing efficiently into the bottom hole-transporting semiconducting layer and preventing them to contribute to the source-drain current.^38^

**Figure 4:**
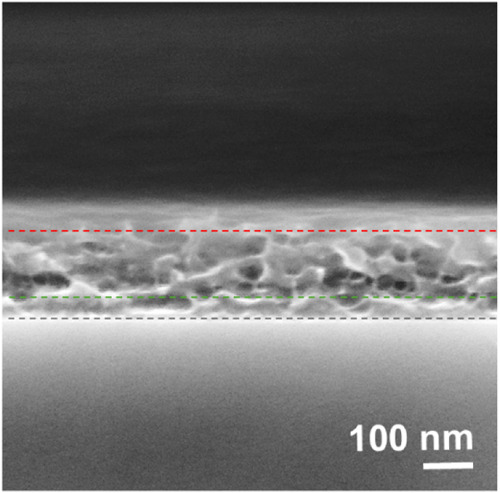
SEM morphology of the DPP-DTT:PyPBTM BHJ thin film with a 1:3 weight ratio on a cross-linked DPP:DTT thin film, deposited onto a silicon substrate. The dashed black line indicates the interface between the silicon substrate and cross-linked DPP:DTT thin film, and the green and red dashed lines indicate the bottom and top interfaces of the DPP-DTT:PyPBTM BHJ thin film with a 1:3 weight ratio.

A more detailed analysis of the best-performing BHJ is provided by SEM imaging of the cross-section of the DPP:DTT/DPP-DTT:PyPBTM BHJ (1:3) stack of the optimized r-OPTs (Figure 4).

Above a continuous film section of about 60 nm thick (attributed to cross-linked DPP-DTT), the thin film section on top comprised between the green and the red dashed lines (attributed to the BHJ) is about 100 nm thick and mostly has a porous morphology. The thickness of the two layers matched well with those measured by profilometry. A similar porous morphology has already been reported in the literature for SEM images of cross sections of polymer/fullerene BHJ films, in particular for annealed BHJs and in the presence of a large fraction of fullerene in the blend (at least 40% by weight).^39−41^ Such porous morphology is a cleaving artifact indicative of selective etching of the small molecular component from the BHJ during the SEM measurements. The voids occupied by PyPBTM molecular aggregates before cleavage have mostly a circular section. This confirms that at a 1:3 weight ratio, PyPBTM forms isolated domains within a continuous matrix of DPP-DTT. Note that the voids are mostly concentrated at the bottom side of the BHJ thin film. This may indicate a gradient of the relative DPP-DTT and PyPBTM concentrations through the thickness of the BHJ film, with the PyPBTM-richer phase close to the bottom interface of the BHJ film in accordance with the literature reporting on the typical vertical segregation occurring in polymer/fullerene BHJ films.^42^

Notably, the r-OPT comprising the DPP-DTT:PyPBTM BHJ with a 1:3 weight ratio behaves as a standard OPT even when the irradiation optical power is varied (Figure S3). By decreasing the irradiation optical power (from 2 to 1 to 0.4 mW/cm^2^), we observed that (i) a gradual lateral shift of the transfer curves, with the threshold voltage gradually moving from positive values toward smaller or negative values, and (ii) a vertical shift of the curve from larger *I*_D_ currents toward smaller *I*_D_ currents. This is due to the gradual decrease of the photovoltaic and photogating effects by decreasing the irradiation optical power.

To provide direct evidence of the electron trapping mechanism at radical sites, on the basis of the good performance of the optimized r-OPT, we extrapolated the electron and hole mobilities of the BHJ with a DPP-DTT:PyPBTM weight ratio of 1:3 from the analysis of the current–voltage curves under dark conditions of charge-selective devices, EODs, and HODs (Figure S4). The electron mobility (μ_N_^SCLC^) and hole mobility (μ_P_^SCLC^) were calculated from the current–voltage curves measured for the EODs and HODs, respectively, fitted by using the Mott–Gourney equation in the SCLC region (see the Experimental Section). The resulting values are compared to those calculated using the same method on a reference BHJ, comprised of DPP-DTT and the standard electron-acceptor PC_61_BM in an optimized weight ratio, as reported in our previous work (Table 2).^35^ μ_N_^SCLC^ in the DPP-DTT:PyPBTM BHJ (1:3) was as low as 3.2 × 10^–8^ cm^2^ V^–1^ s^–1^, which is 100 times lower than that of the standard BHJ based on DPP-DTT:PC_61_BM. On the contrary, μ_p_^SCLC^ in the DPP-DTT:PyPBTM BHJ (1:3) is lower by about 30 times than that of the standard DPP-DTT:PC_61_BM BHJ.

**Table 2:** Bulk Electron Mobility Extrapolated from EOD Devices Correlated with the Photosensitivity of BHJs Based on the Same Donor:Acceptor BHJs

material acceptor	μ_N_^SCLC^ [cm^2^/(V s)]	μ_P_^SCLC^ [cm^2^/(V s)]	μ_P_^SCLC^/μ_N_^SCLC^	*P*
PyPBTM	3.2 × 10^–8^	2.3 × 10^–6^	70	1 × 10^5^
PC_61_BM	4.3 × 10^–6^	7.5 × 10^–5^	17	6.0 × 10^2^

By combining the strong electron affinity of PyPBTM, likely acting on a trapping mechanism as previously demonstrated,^30^ with the optimized morphology of the DPP-DTT:PyPBTM BHJ with a 1:3 weight ratio, the transport of electrons is inhibited while the good mobility of holes is preserved. The ratio between the majority- and minority-carrier mobility, which can be approximated by μ_P_^SCLC^/μ_N_^SCLC^, is about 4 times larger for the DPP-DTT:PyPBTM BHJ than for the standard DPP-DTT:PC_61_BM BHJ (Table 2). This ratio is expected to be proportional to *G* and thus to P in the corresponding r-OPT and OPT. Indeed, the P of the r-OPTs based on the DPP-DTT:PyPBTM BHJ in a 1:3 weight ratio is improved by almost 3 orders of magnitude than the reference OPT based on the standard DPP-DTT:PC_61_BM BHJ (Figure S5 and Table S1), passing from 6.0 × 10^2^ to 1 × 10^5^, respectively.

**Figure 5:**
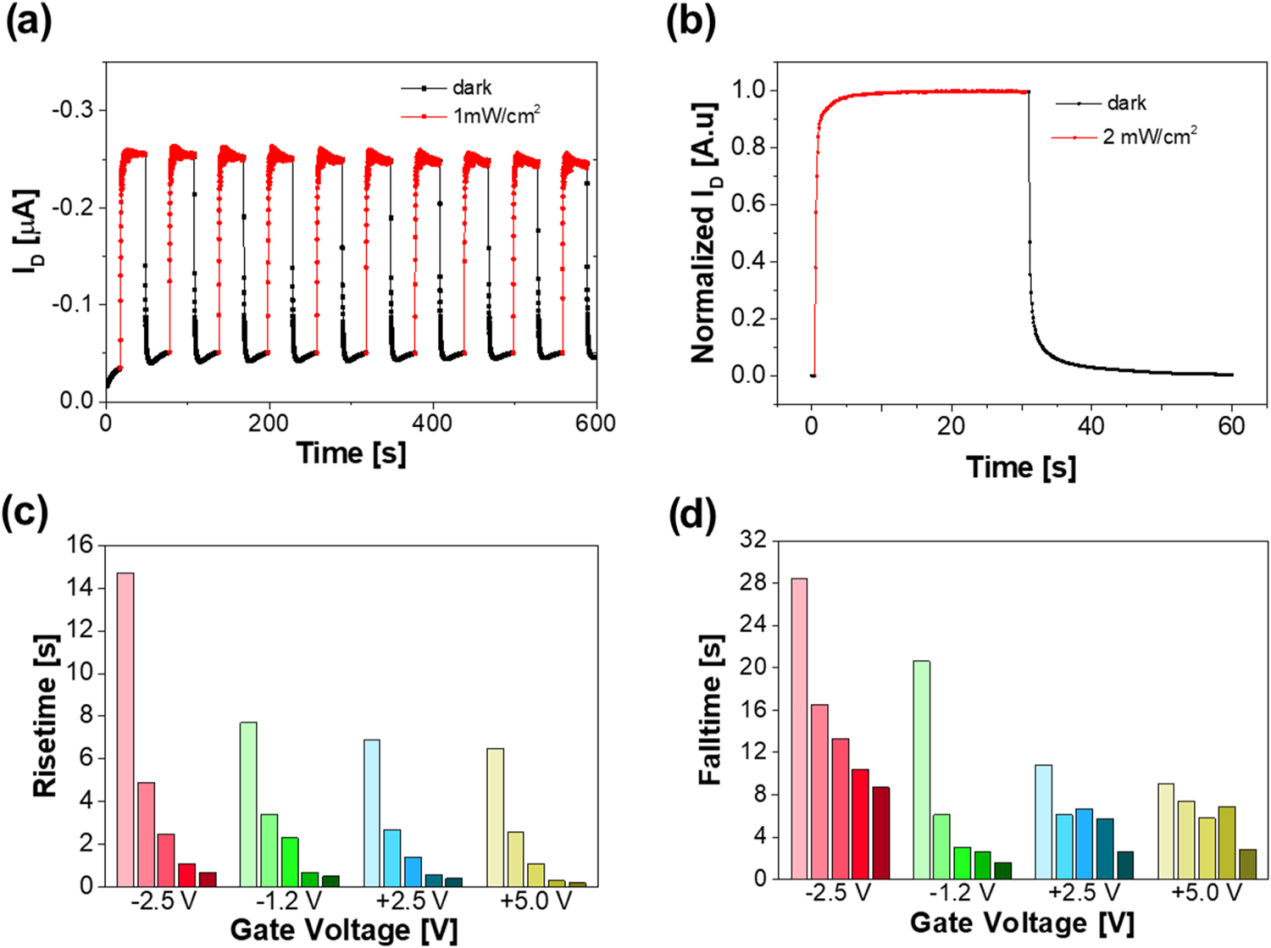
(a) Stability of the r-OPT response to repeated NIR illumination pulses at a power density of 1 mW/cm^2^, within a 10 min time frame. r-OPT is driven at *V*_D_ = −8 V and *V*_G_ = 0 V. (b) r-OPT response to a single NIR illumination pulse at a power density of 2 mW/cm^2^. r-OPT is driven at *V*_D_ = −8 V and *V*_G_ = 0 V. (c, d) Risetime and falltime measured for the r-OPTs at different *V*_G_ values and illumination power densities. In the diagram, the different light intensities used for each *V*_G_ are 0.03, 0.2, 0.4, 1, and 2 mW/cm^2^ and decrease with a decrease of the color shade.

To investigate how the suggested electron trapping by PyPBTM in the BHJ affects the time response of r-OPT, we measured the photocurrent transients during pulsed-light NIR illumination. Note that the remarkable photostability of PyPBTM as open-shell molecules allowed us to register a stable photoresponse from the r-OPTs during 10 min of pulsed illumination.^29^ Indeed, at a repetition rate of 17 mHz and a fixed bias (*V*_G_ = 0 V and *V*_D_ = – 8 V, conditions at which *P* is maximized), the r-OPT shows good on–off switchability and complete dark current recovery when the light is switched off (Figure 5a). The time response of the r-OPT to a single light pulse was measured and calculated at different illumination power densities (from 2 to 0.03 mW/cm^2^) and *V*_G_ biases from −2.5 to 5 V at a fixed *V*_D_ = −8 V(Figure 5c,d). Risetime values were extrapolated from monoexponential fitting, and falltimes were extrapolated from biexponential fitting as amplitude-averaged values.

By increasing *V*_G_ toward more positive values and increasing the illumination optical power density, the r-OPT response becomes faster, as expected from the more efficient recombination of opposite charges and/or the detrapping of electrons in the BHJ. Indeed, the fastest response is registered at *V*_G_ = 5 V and at an illumination power density of 2 mW cm^–2^. The time response of the r-OPT to a single light pulse under these conditions is shown in Figure 5b, from which a risetime of 1.1 s and a falltime of 5.8 s are extrapolated. Even if the risetimes are consistent with those reported for similar OPTs,^43,44^ the long falltime values could represent a limit for possible applications of OPTs in optical communications or image recording, where devices typically need to return quickly to the initial current level before the next optical pulse. However, the possibility to control the electron trapping/detrapping process at radical sites, thus the time response of the device, by simply acting on the *V*_G_ potential, opens the way to the exploitation of r-OPTs for other intriguing and innovative applications of organic optoelectronic devices, such as neuromorphic applications, as demonstrated for similar devices.^45^

Overall, the performance of the r-OPTs presented here is in line with most of the highly performing solution-processed BHJ OPTs operating in the NIR.^46^ In this regard, in Table 3, we compare the most relevant figures of merit of the presented optimized r-OPTs with those of other OPTs reported in the literature, based on a BHJ NIR light-sensing layer. By comparing the performance within OPTs with a BHJ photoactive layer composed of a DPP-DTT hole donor and different electron acceptors, the largest P is obtained for our r-OPTs based on the organic persistent radical as the electron acceptor, instead of other well-assessed electron-accepting moieties such as PC_61_BM,^32^ PC_71_BM,^38^ or the most common nonfullerene acceptor ITIC.^47^ The largest *P* is obtained for our r-OPTs also in comparison to other BHJ light-sensing layers, based on different polymers and nonfullerene acceptors, such as phthalocyanine derivative electron acceptors and PTCDA.^7^ Evidently, the use of tailored and creative fabrication protocols, for decoupling^48^ or engineering the interfaces between the field-effect charge-transporting layer and the BHJ light-sensing layer^49^ or between the field-effect charge-transporting layer and the gate dielectric^50^ can lead to an improved P with respect to the present work, at the expenses of increased complexity in the fabrication process.

**Table 3:** Comparison of the Performance of the r-OPT Presented in this Work with Other NIR-Sensitive OPTs with a BHJ Light-Sensing Layer

OPT active layer	μ_P_^FET^ [cm^2^/(V s)]	*P*	*R* [A/W]	refs
DPP-DTT:PC_61_BM	3 × 10^–1^	1.6 × 10^4^	5 × 10^5^ (@810 nm)	(32)
CuPc/PTCDA:PbPc	1.2 × 10^–3^	9.4 × 10^2^	3.2 × 10^–1^ (@808 nm)	(7)
DPP-DTT:PC_61_BM	ND	∼2 × 10^5^	3.5 × 10^5^ (@810 nm)	(50)
P3HT/PMMA/PEHTPPD-BT (gate-sensing layer configuration)	∼5	ND	3.4 × 10^–1^ (@780 nm)	(48)
PDPP3T/PDPP3T:PC_61_BM (with solvent vapor annealing)	7.5 × 10^–2^	1.7 × 10^7^	8.7 × 10^3^ (@850 nm)	(49)
DPP-DTT:PC_71_BM	2 × 10^–1^	8 × 10^3^	3.2 × 10^1^ (@870 nm)	(38)
DPP-DTT:ITIC	1	10^1^	2.3 × 10^1^ (@750 nm)	(47)
DPP-DTT/DPP-DTT:PC_71_BM	3.3 × 10^–2^	2 × 10^2^	2 × 10^–1^ (@770 nm)	(35)
DPP-DTT/DPP-DTT:PC_61_BM	6.1 × 10^–2^	1.2 × 10^3^	2 × 10^–1^ (@770 nm)	(35)
DPP-DTT/DPP-DTT:PyPBTM	2.1 × 10^–2^	3.5 × 10^5^	1.1 × 10^–1^ (@ 770 nm)	this work

## Conclusions

In this work, we report for the first time the direct implementation of an organic persistent radical (PyPBTM) as an effective electron acceptor in a BHJ-based bilayer OPT fully processed from the solution. The bilayer architecture is based on solution-processed and solvent-resistant hole-transporting semiconducting and dielectric polymeric layers. This allowed us to test different solution-processed BHJ photoactive layers based on different ratios of the electron acceptor (PyPBTM) and electron donor (the low band gap polymer DPP-DTT). Investigations of the optical, electronic, and morphological characteristics of DPP-DTT:PyPBTM BHJ films with donor:acceptor ratios of 1:2, 1:3, and 1:4 allowed the selection of the BHJ with the best characteristics before incorporation into OPTs. The BHJ photoactive film with a 1:3 DPP-DTT:PyPBTM ratio combined together the strong electron affinity and electron-trapping capability of PyPBTM with a morphology prone to inhibit the mobility of minority carriers (electrons) and to allow diffusion of majority carriers (holes). The best light-sensing performance was registered indeed for the OPT comprising the BHJ photoactive film with a 1:3 DPP-DTT:PyPBTM ratio, reaching state-of-the-art *P* as high as 1 × 10^5^, and a D* of 1.9 × 10^13^ Jones, under NIR illumination at 770 nm with an optical power density of 2 mW/cm^2^. The performance of the OPT based on the 1:3 DPP-DTT:PyPBTM BHJ is improved compared to the analogous OPT comprising a BHJ with the same DPP-DTT and PC_61_BM as the standard electron acceptor, owing to an increased photogain, likely due to a trapping mechanism at the PyPBTM sites. A trapping mechanism is also likely responsible for the time response in the second range of the presented OPTs. However, we also demonstrated that the time response of the OPT can be effectively modulated and shortened by acting on the gate bias, thus controlling the electron trapping/detrapping process that occurs in the photoactive BHJ. The present work opens the way for the application of a new class of organic molecular acceptors in BHJ-based OPTs, such as organic persistent radicals, for a plethora of applications, including memory and neuromorphic optoelectronic devices and circuits.

## Supporting Information Available

Absorption spectra of the films with different DPP-DTT and PyPBTM weight ratios; schematic structure of the reference OPT; performance of r-OPTs at different illumination optical powers; schematic energy level diagram and device structures of HODs and EODs; schematic structure and optoelectronic performance of the reference OPT with DPP-DTT:PC_61_BM as the BHJ photoactive layer (PDF)
