# Supplementary material for: High-Sensitivity Solution-Processed Organic Phototransistor Based on a Bulk Heterojunction with a Persistent Radical as the Electron Acceptor
Source: ACS Appl Electron Mater. 2025 Apr 23;7(9):3694–703. doi: 10.1021/acsaelm.4c02334 (PMC12080257; doi:10.1021/acsaelm.4c02334)
Supplement: Supplementary file 1 — el4c02334_si_001.pdf [file el4c02334_si_001.pdf]

Supporting Information

High sensitivity solution-processed organic phototransistor based on a bulk heterojunction with a persistent radical as the electron acceptor

Giulia Baroni,[a‡] Francesco Reginato,[a‡] Sara Mattiello,[b] Salvatore Moschetto,[a] Mario Prosa,[a] Margherita Bolognesi,[a] Luca Beverina,[b] Stefano Toffanin[a]**

[a] Institute of Nanostructured Materials (ISMN) – National Research Council (CNR), Via P. Gobetti 101, Bologna 40129, Italy; margherita.bolognesi@cnr.it; stefano.toffanin@cnr.it

[b] Department of Materials Science, University of Milano-Bicocca, Via R. Cozzi 55, Milano, Italy

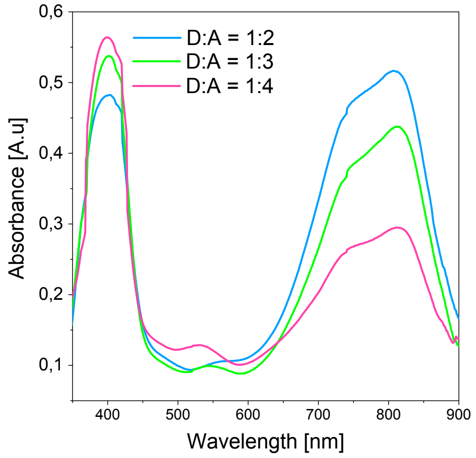

Figure S1. Absorption spectra of the tested different DPP-DTT and PyPBTM weight ratios i.e. 1:2, 1:3 and 1:4.

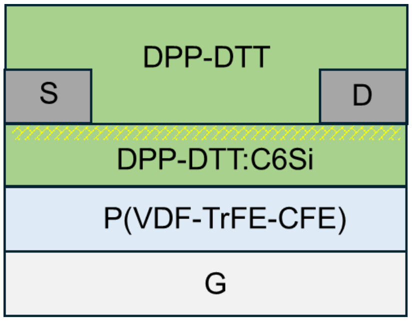

Figure S2. reference OPT having the bilayer architecture with the acceptor-free photoactive layer, composed of pristine DPP-DTT.

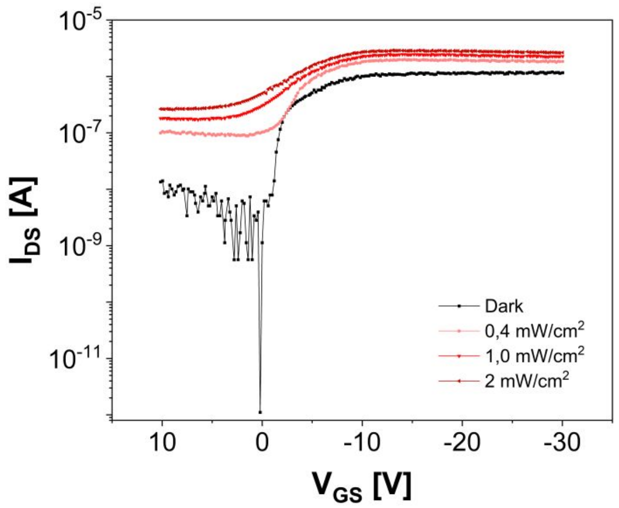

Figure S3 Transfer curves of the OPTs based the DTT:PyPBTM 1:3 BHJ at V_D =-8 V, in the dark and under illumination in the NIR, at 770 nm, with variable optical power of 2 mW/cm^2, 1 mW/cm^2 and 0,4 mW/cm^2.

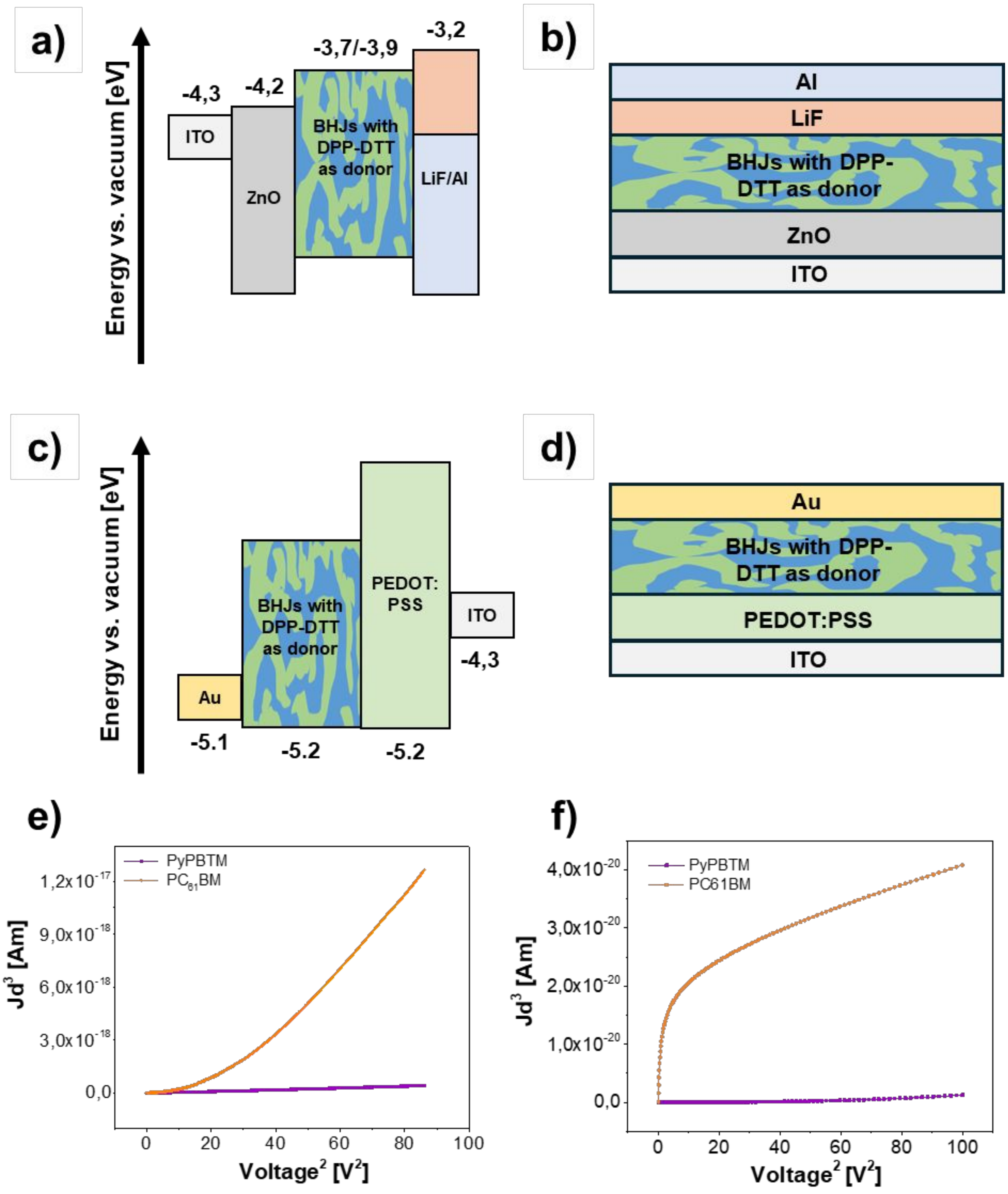

Figure S4. (a) Schematic energy level diagram and (b) schematic device structure of EODs. (c) Schematic energy level diagram and (d) schematic device structure of HODs. (e) Jd^3-V curves measured for HODs. (f) Jd^3-V curves measured for EODs.

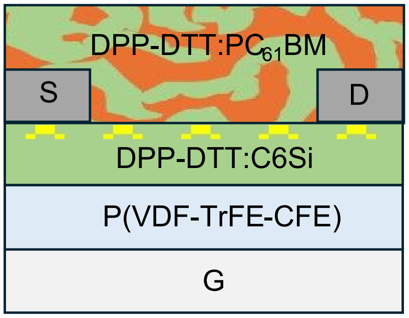

Figure S5. Schematic device structure for a OPT with DPP-DTT:$PC_{61}BM$ as standard BHJ

Table S1. Summary of the FOMs of the OPT with the standard DPP-DTT:$PC_{61}BM$ BHJ.

DPP-DTT:$PC_{61}BM$	μ_P^{FET} [a)] [$cm^2/V\cdot s$]	V_{TH} [a)] [V]	P [b)]	R [b)] [A/W]	D* [b)] [Jones]
1:1	$5{,}0 \times 10^{-2}$	-3,0	600	0,4	7×10^{-11}

[a)] calculated from transfer curves in the saturation regime in dark condition; [b)] calculated from transfer curves at V_{DS}= - 8V, best results are reported.
